# Hemodynamic Effects of Stent Struts versus Straightening of Vessels in Stent-Assisted Coil Embolization for Sidewall Cerebral Aneurysms

**DOI:** 10.1371/journal.pone.0108033

**Published:** 2014-09-23

**Authors:** Kenichi Kono, Aki Shintani, Tomoaki Terada

**Affiliations:** Department of Neurosurgery, Wakayama Rosai Hospital, Wakayama, Japan; Coastal Carolina University, United States of America

## Abstract

**Background:**

Recent clinical studies have shown that recanalization rates are lower in stent-assisted coil embolization than in coiling alone in the treatment of cerebral aneurysms.

**Objective:**

This study aimed to assess and compare the hemodynamic effect of stent struts and straightening of vessels by stent placement on reducing flow velocity in sidewall aneurysms, with the goal of reducing recanalization rates.

**Methods:**

We evaluated 16 sidewall aneurysms treated with Enterprise stents. We performed computational fluid dynamics simulations using patient-specific geometries before and after treatment, with or without stent struts.

**Results:**

Stent placement straightened vessels by a mean (±standard deviation) of 12.9°±13.1° 6 months after treatment. Placement of stent struts in the initial vessel geometries reduced flow velocity in aneurysms by 23.1%±6.3%. Straightening of vessels without stent struts reduced flow velocity by 9.6%±12.6%. Stent struts had significantly stronger effects on reducing flow velocity than straightening (P = 0.004, Wilcoxon test). Deviation of the effects was larger by straightening than by stent struts (P = 0.01, F-test). The combination of stent struts and straightening reduced flow velocity by 32.6%±12.2%. There was a trend that larger inflow angles produced a larger reduction in flow velocity by straightening of vessels (P = 0.16).

**Conclusion:**

In sidewall aneurysms, stent struts have stronger effects (approximately 2 times) on reduction in flow velocity than straightening of vessels. Hemodynamic effects by straightening vary in each case and can be predicted by inflow angles of pre-operative vessel geometry. These results may be useful to design a treatment strategy for reducing recanalization rates.

## Introduction

Stent-assisted coil embolization (SACE) is widely accepted for endovascular treatment of wide-neck or complex cerebral aneurysms. Several recent reports have shown that SACE promotes occlusion of incompletely coiled aneurysms and lowers recanalization rates compared with coiling alone [1]–[7]. This situation probably occurs because of two types of hemodynamic effects by stent placement: stent struts and straightening of vessels. Hemodynamics effects by stent struts have been widely studied by computational fluid dynamics (CFD) simulations and *in vitro* experiments [8]–[12]. Stent placement straightens parent vessels [13], [14]. Hemodynamic studies on straightening of vessels have also been reported [15], [16]. However, there are no studies on hemodynamic effects of stent struts and straightening combined.

In this study, we performed CFD simulations of 16 patient-specific sidewall aneurysms treated with SACE. We determined the main factor for reducing flow velocity in aneurysms and recanalization rates. Our results may provide helpful information to reduce recanalization rates in clinical practice.

## Methods

The Institution Review Board of Wakayama Rosai Hospital approved this retrospective study. The requirement for informed consent was waived. Patient records and geometric data were anonymized prior to analysis. All analyses were performed only by two authors (KK and AS), who were also engaged in clinical cases as neurosurgeons.

### Clinical Cases

There were 49 consecutive cases of aneurysms treated by SACE using Enterprise stents (Cordis Neurovascular, Miami, FL, USA) between July 2010 and October 2013 in our institution. Among these, there were 16 sidewall aneurysms without any branches arising from the neck of the aneurysms. We included these 16 cases in this study (Cases 1–16). All of the 16 aneurysms were unruptured vertebral artery aneurysms and treated by a single stent. The mean (standard deviation) age was 58.4±9.7 years. The mean maximum diameter of the aneurysms was 8.2±2.0 mm. The mean diameters of the proximal and distal parent vessels were 3.6±0.7 mm and 3.2±0.8 mm, respectively. In the mean follow-up period of 27±11 months, recanalization occurred in one case (6.3%, Case 6), and retreatment was performed.

The rest of the 33 cases were excluded because of the following reasons: insufficient imaging quality for CFD (n = 2), retreatment cases (n = 5) [17], [18], post-operative in-stent stenosis (n = 1), post-operative in-stent occlusion (n = 1) [19], bifurcation aneurysms including aneurysms with small branches arising from the neck of aneurysms, such as those at the junction of the vertebral artery and the posterior inferior cerebellar artery, or ophthalmic artery aneurysms (n = 17) [20], and cavernous carotid artery aneurysms (n = 7) [21]. Cavernous carotid artery aneurysms were excluded because the internal carotid artery around the cavernous portion is partly covered with skull bones, which disturb changes in vascular geometry by stent placement.

### Reconstruction of Geometry

**Figure 1:**
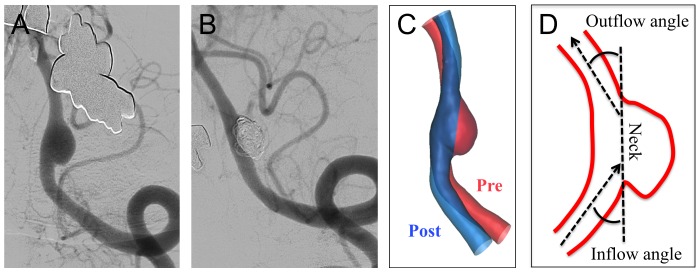
Angiograms and illustrations of straightening of vessels by stent placement in Case 1. (**A**) An angiogram shows a sidewall aneurysm at the left vertebral artery. (**B**) Six months after stent-assisted coil embolization for the aneurysm, an angiogram shows complete occlusion of the aneurysm and straightening of the vessel. (**C**) Two 3D images of the vessel and the aneurysm at pre- (in red) and post-treatment (in blue) are fused. (**D**) The “inflow angle” and “outflow angle” are defined as angles between the neck plane of an aneurysm and an inflow line or an outflow line, respectively. The angles can be negative when the angles show opposite directions.

Three-dimensional images of vascular geometry before and 6 months after stent placement were obtained by 3D rotational angiography. Using an engineering design software, 3-matic (Version 7.0; Materialise NV, Leuven, Belgium), the two images were fused as previously reported [22], [23]. We used a three-point registration algorithm of the 3-matic software by choosing three points located at similar regions on both 3D images. We then performed manual registration based on the geometry of the aneurysm and a parent vessel near the aneurysm (Figure 1A–C). Because of artifacts of coils, the post-operative 3D models were created by fusion of the pre-operative 3D aneurysm geometry and the post-operative straightened vessel geometry (Figure 1C). The aneurysm geometry was the same in both pre- and post-operative models, which assured valid evaluation of changes in flow velocity in the aneurysm by stent struts or straitening of vessels. Inflow angles and outflow angles were defined as shown in Figure 1D. In the pre- and post-operative models, these angles were measured in the 2D view, in which the neck of an aneurysm, an inflow line, and an outflow line could be observed on the same 2D plane (Figure 1C and D). Measurements of these angles were performed by two operators (KK and AS) independently, and mean values were used.

**Figure 2:**
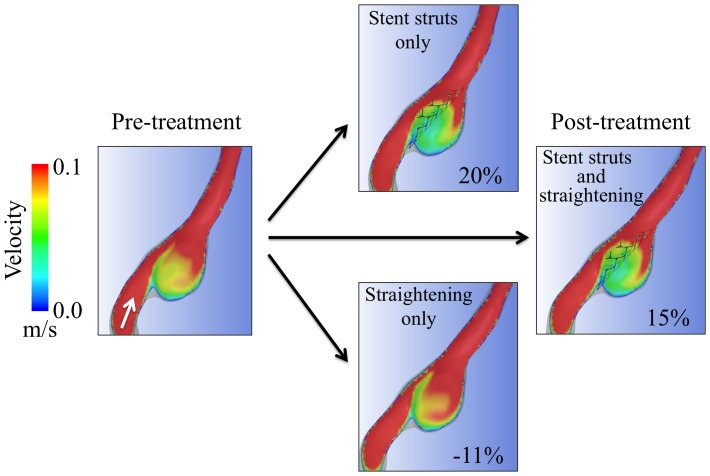
Illustrations of hemodynamic analysis of reduction in flow velocity in an aneurysm of Case 1. Contours of the cut plane are colored according to mean flow velocity. The white arrow in the pre-treatment image indicates the flow direction. Flow velocity was reduced by 20% after placement of stent struts on the initial geometry. Flow velocity was reduced by −11% (i.e., increased by 11%) after straightening of a vessel without stent struts. The combination of both stent struts and straightening resulted in a flow reduction of 15%. Straightening of the vessel changed the inflow angle from −24° to −15°.

To obtain the stent geometry, a 4.5 mm×28 mm Enterprise stent was scanned by micro-CT, using the Toscaner-30900 µC3 (Toshiba IT & Control Systems Corp., Tokyo, Japan). The resolution of the micro-CT scanner is 5 µm. Images were obtained in the standard triangulated language format. The width of each strut of the stent obtained by micro-CT was 0.0794±0.005 mm (n = 50; 95% confidence interval: 0.0779–0.0810 mm). The accuracy of the stent geometry obtained by micro-CT was sufficient because the width of the strut of the Enterprise stent is 0.078 mm [24]. This stent geometry was used to cover the neck of the aneurysm. The stent struts were manually fitted on the neck orifice of each aneurysm (Figure 2). To reduce computer resources, we did not place the stent struts in other regions of parent vessels. Although we used Enterprise stents with different lengths in clinical cases, all Enterprise stents have the same diameter (4.5 mm). Geometry of stent struts of any Enterprise stent is identical, and the geometry of a 28-mm Enterprise stent was sufficient to create aneurysm models with stents. In these processes, we created four 3D models in each case: pre-treatment models with or without stent struts and post-treatment models (i.e., straightened vessel) with or without stent struts (Figure 2). To examine reproducibility of these processes of 3D geometry reconstruction, four models were created from the initial geometries in Case 1 for three times. These processes were preformed by a single operator (KK). To examine operator independency of these processes, a different operator (AS) independently created four models in Case 1.

### CFD Simulations

We performed CFD simulations in a similar manner as described previously [22], [25]. The fluid domains were extruded at the inlet to allow fully developed flow and meshed using ICEM CFD software (Version 14.5, Ansys Inc., Canonsburg, PA, USA) to create finite volume tetrahedral elements. The smallest grid size was 0.025 mm, which was approximately 1/3 of the width of the strut of the Enterprise stent (0.078 mm). This is sufficient to obtain global flow patterns in aneurysms [26]. Small meshes were generated near the stent struts and large meshes were generated far from the stent struts to enhance local resolution, while keeping the total number of elements within reasonable bounds. The number of elements in each model was approximately 2,200,000–3,100,000, which was confirmed to be adequate to calculate velocity by creating meshes of finer grid densities. Approximately doubled grid densities showed <2% differences in mean velocity in the aneurysm, and grid independence was confirmed. We did not include coils in these simulations because of technical difficulties as well as most of previous CFD studies on stents for cerebral aneurysms [8], [9]. Blood was modeled as a Newtonian fluid with a density of 1056 kg/m^3^ and a viscosity of 0.0035 kg/m·s. A rigid-wall no-slip boundary condition was implemented at the vessel walls. We performed pulsatile flow simulations with an implicit solver, Ansys CFX (Version 14.5, Ansys Inc.), the accuracy of which has been validated previously [25], [27]. For the inlet flow conditions, we used the volumetric flow rate waveform of the vertebral artery of normal subjects as reported by Ford et al [28]. The flow rate was scaled so that cycle-averaged wall shear stress at the parental artery would be 1.5 Pa. [29] Zero pressure was imposed at the outlets. The width of the time step for calculation was set at 0.005 s. A smaller time step, 0.001 s, showed <0.2% difference in mean flow velocity in aneurysms, assuring verification of a time step of 0.005 s. Calculations were performed for three cardiac cycles, and the result of the last cycle was used for analysis. We examined the volume-averaged and cycle-averaged flow velocity in aneurysms in four different models in each case (Figure 2). Flow reduction ratio is defined as (V_pre – V_post)/V_Pre where V_pre is flow velocity before treatment and V_post is flow velocity after placement of stent struts only, straightening of vessels only, or both of them (Figure 2). We defined the flow reduction ratio as a normalized parameter of reduction in flow velocity (V_pre – V_post), because flow velocity in aneurysms before treatment (V_pre) was different in each case.

### Statistical Analysis

Statistical analysis was performed using SPSS version 20 (IBM Corp., Armonk, NY, USA). The Wilcoxon test, F-test, or Spearman's correlation analysis was used. The level of significance was set at P<0.05. Statistical data are described as mean ± standard deviation.

## Results

The study of reproducibility of geometry reconstruction by repeating reconstruction for three times in Case 1 showed that flow reduction ratios were 20.3%, 21.2%, and 20.8% by stent struts, −11.4%, −10.8%, and −12.1% by straightening, and 14.9%, 15.7%, and 14.7% by both. The study of operator independency showed that the flow reduction ratios were 20.3% and 22.5% by straightening, −11.4% and −12.2% by stent struts, and 14.9% and 15.8% by both, in operators 1 and 2, respectively. In both studies, absolute values of differences of flow reduction ratios were within 2.2%. These results assured reasonable reproducibility and operators' independency in the reconstruction processes of 3D models.

The mean pre- and post-operative angles (inflow angle + outflow angle) were 61°±46° and 49°±37°, respectively. Stent placement straightened vessels by 12.9°±13.1° 6 months after treatment.

**Figure 3:**
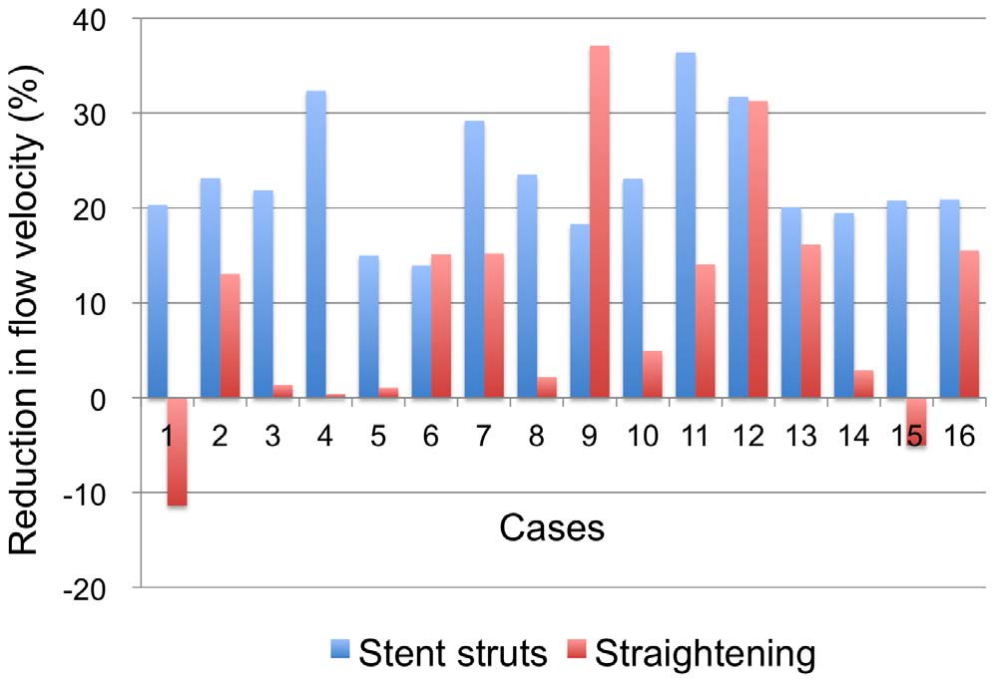
Reduction in flow velocity in 16 cases by either stent struts or straightening of vessels.

An example of the results of CFD simulations of Case 1 is shown in Figure 2. A summary of CFD results of 16 cases is shown in Figure 3. The flow velocity in 16 aneurysms was 35.1±18.2 cm/s before treatment, 27.3±15.1 cm/s after placement of stent struts only, 31.5±17.2 cm/s after straightening of vessels only, and 23.8±14.4 cm/s after both of placement of stent struts and straightening of vessels. Reduction ratios in flow velocity by stent struts and straightening of vessels were 23.1%±6.3% (range: 13.9–36.4%) and 9.6%±12.6% (range: −11.4% to 37.1%), respectively. The effects from stent struts were significantly larger than those from straightening of vessels (P = 0.004, Wilcoxon test). Deviation of the effects was larger by straightening than by stent struts (P = 0.01, F-test). Therefore, flow reduction ratios by stent struts were relatively constant, while those by straightening of vessels varied in each case (Figure 3). The combination of stent struts and straightening of vessels reduced flow velocity by 32.6%±12.2% (range: 14.0–54.9%). In the recanalized case, Case 6, the flow reduction ratio in the aneurysm was 13.9%, 15.1%, and 28.1% by stent struts, straightening, and both of them, respectively. This ratio (28.1%) was slightly below the mean value (32.6%).

**Figure 4:**
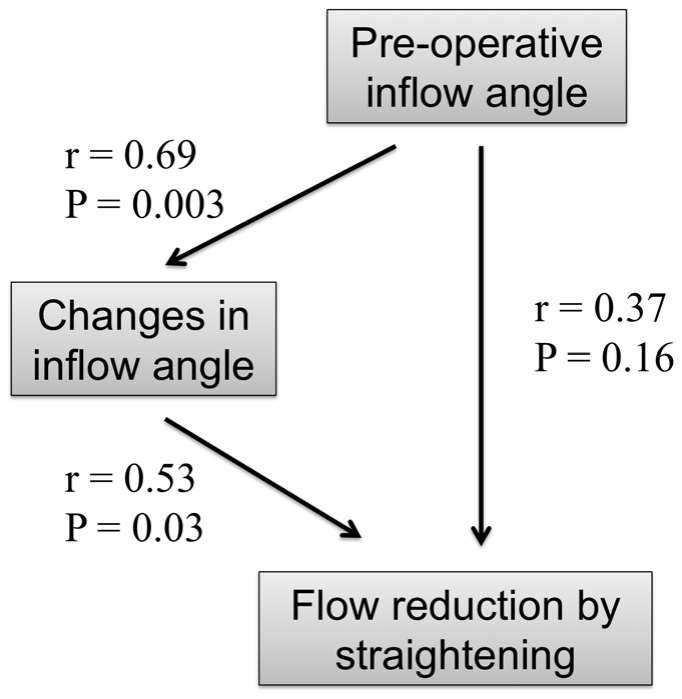
Correlations among pre-operative inflow angle, changes in inflow angle, and flow reduction by vessel straightening.

An increase in flow velocity in the aneurysms by straightening was observed in two cases, while flow velocity was reduced in all of the cases by stent struts (Figure 3). In these two cases, pre-operative inflow angles were negative (e.g., Figure 2). To further investigate variability of flow reduction by straightening of vessels, we focused on relationships among pre-operative inflow angles and changes in inflow angles by stent placement (Figure 4). There were positive correlations between pre-operative inflow angles and changes in inflow angle (r = 0.69, P = 0.003, Spearman's correlation), changes in inflow angle and flow reduction by straightening (r = 0.53, P = 0.03), and pre-operative inflow angle and flow reduction by straightening (r = 0.37, P = 0.16). Therefore, larger pre-operative inflow angles changed inflow angles more by stent placement, and resulted in more flow reduction by straightening of vessels.

## Discussion

### Clinical Aspects of SACE

An important issue of coil embolization of aneurysms is how to decrease recanalization rates because recanalization may require retreatment, or even cause subarachnoid hemorrhage. Several recent reports have shown that SACE promotes occlusion of incompletely coiled aneurysms [2]–[7] and significantly lowers recanalization rates compared with coiling alone (14.9% vs. 33.5% [3], 8.1% vs. 37.5% [2], and 17.2% vs. 38.9% [1]). This situation probably occurs because of the hemodynamic effects of stent struts and straightening of vessels by stent placement. There are a few reports on straightening of vessels by stent placement. [13], [14] Vascular angles change by a mean of 30° in anterior communicating artery aneurysms [14] and by a mean of 36° in bifurcation aneurysms. [13] Vessel angles change more in vessels with smaller diameters or more acute bending vessels, and Enterprise stents change vessel angles more than Neuroform stents (Stryker Neurovascular, Freemont, CA, USA). [13] In our series, vessel angles straightened by a mean of 12.9°, which is lower than that in these previous reports. The reason for this difference between studies is probably because the vessel diameter of the vertebral artery is larger than that of the anterior cerebral artery or the middle cerebral artery. In addition, these previous reports examined bifurcation aneurysms, while our study examined sidewall aneurysms. Angles of parent vessels at aneurysms are usually more acute in bifurcation aneurysms than in sidewall aneurysms, which could explain smaller changes in vessel angles in our series than in these previous reports.

Low packing density is associated with recanalization in coiling alone in treatment of aneurysms, [30] and packing densities of at least 20–25% are needed to protect against recurrence [31], [32]. These results suggest that reduction in flow velocity in aneurysms is important to prevent recanalization. In SACE, there are no significant differences in recurrence between moderate (12–22%) and high (>22%) packing density [33]. Meta-analysis of SACE versus coiling alone showed a mean packing density of 27.4% in the SACE group compared with 28.2% in the coiling only group (1474 patients in total) [7]. These results suggest that packing density does not increase by stent placement, but that SACE with lower or equivalent packing density can achieve higher occlusion rates and less recanalization rates than coiling alone. Therefore, we presumed that hemodynamic effects, not packing density, by both stent struts and straightening of vessels are associated with a reduction in recanalization rates, and designed the current hemodynamic study.

### Hemodynamics Effects Caused by Stent Placement

In our study, stent struts had relatively constant effects on reducing flow velocity by approximately 23%. Straightening of vessels had variable effects on reducing flow velocity, with a mean reduction of approximately 10%, which is half that of the effects by stent struts. The combination of both stent struts and straightening reduced flow velocity by a mean of approximately 33%. These results show that combination effects are approximately the sum of the two effects (i.e., 23%+10% = 33%). Therefore, hemodynamic effects caused by straightening of vessels should be taken into account in hemodynamic studies of stent placement, even though the effects are half of those by stent struts. In the recanalized case, Case 6, stent placement showed a 28.1% flow reduction ratio, which is a slightly below the mean flow reduction ratio of 33%. Therefore, we could not explain recanalization in this particular case by the flow reduction ratio in the aneurysm. However, we consider that a mean of 33% in flow reduction could explain the low recanalization rates in our series (6.3%), as well as the low rates in previously reported series of SACE [1]–[3].

Hemodynamics of several configurations of stent placement for aneurysms have been previously studied [8]–[12]. All of these studies evaluated hemodynamic effects of stent struts with unchanged vessel geometries. Tremmel et al. reported that single Enterprise stent placement reduces mean velocity by approximately 15% in a single basilar trunk aneurysm because of the stent struts [9], which is within the range of our results. This supports accuracy of our CFD studies with manual stent placement. Although it would be better to obtain stent geometry in patient-specific silicone models by micro-CT for accurate CFD simulations [8], [34], this would be unrealistic for the 16 cases. Virtual deployment of stents would be better for more accurate stent geometry than manual placement of stents [35], [36]. However, we consider that the process of our manual stent placement was acceptable in this study because we showed reproducibility and operator independency of the process, and obtained similar reduction ratios in flow velocity compared with other studies [9].

Two hemodynamic studies of straightening of vessels have been reported [15], [16]. Both studies used vessel geometries without aneurysms by removing aneurysms from original geometries. In single stent-assisted coiling for bifurcation aneurysms, changes in vessel angles by stent placement decrease pressure at the bifurcation apex by 2.3 Pa (0.02 mmHg) and decrease the width of the flow impingement zone between two peaks of wall shear stress by 0.9 mm [16]. In Y-stent coiling of basilar bifurcation aneurysms, angular remodeling leads to significant narrowing of the wall shear stress interpeak at the apex, redirecting high wall shear stress away from the neck transition zone with the native vessel toward the inert coil mass [15]. These results are difficult to interpret because it is unclear whether these changes in pressure and wall shear stress cause a reduction in recanalization rates, while a reduction in flow velocity in our study may be more directly associated with a reduction in recanalization rates. In addition, no previous studies have addressed the importance of vessel straightening compared with hemodynamic effects caused by stent struts. Therefore, in our study, we addressed this issue.

Our results of correlations among pre-operative inflow angle, changes in inflow angle, and flow reduction by straightening were as expected (Figure 4). Stents tend to straighten acute bending vessels more than relatively straight vessels [13]. Larger changes in inflow angles reduce flow velocity more in aneurysms. Therefore, larger inflow angles reduce flow velocity more in aneurysms by straightening of vessels. These results provide helpful information for predicting hemodynamic effects of straightening by stent placement using inflow angles of pre-operative vessel geometry. If the inflow angle is large, flow reduction can be maximized by straightening of a vessel using a longer Enterprise stent rather than a shorter Neuroform stent. This can be achieved because Enterprise stents change vessel angles more than Neuroform stents and longer stents change these angles more than shorter stents [13]. However, there are cases where straightening increases flow velocity in an aneurysm, such as in Case 1 (Figure 2). This can be predicted by negative pre-operative inflow angles. Therefore, if pre-operative inflow angles are negative, a shorter Neuroform stent may be better for not increasing flow velocity in an aneurysm than a longer Enterprise stent. These “pre-operative inflow angle”-based treatment strategies may help to reduce recanalization rates. Future prospective large-scale studies will be necessary to test this hypothesis.

Hemodynamic effects by stent struts are relatively constant compared with those by straightening of vessels. Stents with lower porosity cause a greater decrease in flow velocity in aneurysms. Therefore, Enterprise closed-cell stents with lower porosity may be more effective for reducing flow velocity in aneurysms and recanalization rates than Neuroform open-cell stents with higher porosity. This strategy is supported by a report, which showed that closed-cell stents are associated with significantly lower recanalization rates in a study of 508 cases treated with SACE [4].

In clinical practice, in addition to recanalization rates, properties of open- or closed-cell stents, such as ease of delivery, stability, and vessel wall apposition, should be considered [37]. Therefore, treatment strategies based on inflow angles and stent porosity for reducing recanalization rates are only one aspect to consider in clinical practice. However, in cases of recanalized aneurysms or aneurysms that are likely to be recanalized, these treatment strategies may be helpful.

Most CFD studies on stents for aneurysms, including our study, did not include coils in simulations because of technical difficulties [8], [9]. Although packing density of coils is another factor for recanalization, we commonly try to insert coils in aneurysms as many as possible. In such situations, current results of CFD simulations of stent placement without coils are still valid and helpful. Our intention in this study was not to recommend stent-only therapy, but to demonstrate that stent therapy with coils reduces recanalization rates by stent struts and straightening of vessels.

### Limitations of the Study

In our CFD study, we simplified several properties, such as the viscoelasticity of the vessel wall, the non-Newtonian property of the blood, and non-patient-specific inlet conditions. Although these simplifications may cause differences between the results of CFD simulation and the *in vivo* state, the main hemodynamic features are thought to be preserved [38]. Patient-specific inlet conditions are necessary for accurate calculations of hemodynamic parameters [39], [40]. However, we evaluated reduction ratios in flow velocity in each case, and this comparison within the same case may be relatively robust to different conditions of simulations. The stent struts were manually fitted on the neck orifice of each aneurysm. Geometry of stent struts depends on diameter and curvature of parent vessels and the way of stent delivery such as a push-pull technique [41], [42]. Although structural simulations of stent deployment would better [36], [43], we do not have such techniques to perform them. At least, as mentioned in the discussion section, reduction rates of flow velocity in our case series are comparable to previous studies, demonstrating that our methodology and results are sufficient to lead to conclusions.

The number of cases in this study was relatively small. However, we observed statistical differences and clarified characteristics of hemodynamic effects by stent struts and vessel straightening. Because our cases were all vertebral artery aneurysms, the results may not be applicable to aneurysms at other locations. In particular, when vessel angles change more than those in the current series because of small diameters of parent vessels, hemodynamic effects by straightening of vessels might increase and outweigh those by stent struts. More cases including aneurysms at other locations are required to address these issues.

We only examined sidewall aneurysms because we consider that hemodynamic features are different between sidewall and bifurcation aneurysms, and that hemodynamic studies should be performed separately. We are planning to perform the same type of CFD studies on bifurcation aneurysms in the future. In addition, sidewall aneurysms can be treated by flow diverter stents. However, in treatments by flow diverters, there are concerns of delayed rupture, possibility of incomplete occlusion of aneurysms, and occlusion of perforators especially at posterior circulations [44], [45]. Flow diverters have yet to be replaced with high porosity stents even for sidewall aneurysms. Therefore, this study is still helpful for clinicians to design treatment strategy.

## Conclusions

This is the first report to compare hemodynamic effects caused by stent struts and straightening of vessels in SACE. In sidewall aneurysms, stent struts have approximately twice as strong effects on reduction of flow velocity than straightening of vessels. Hemodynamic effects caused by straightening of vessels vary in each case and can be predicted by inflow angles of pre-operative vessel geometry. These results may be useful for designing treatment strategies to reduce recanalization rates.

## References

[1] Chalouhi N, Jabbour P, Gonzalez LF, Dumont AS, Rosenwasser R, et al (2012) Safety and Efficacy of Endovascular Treatment of Basilar Tip Aneurysms By Coiling With and Without Stent-Assistance: A Review of 235 Cases. Neurosurgery 71: 785–794. 22743359 10.1227/NEU.0b013e318265a416

[2] Lawson MF, Newman WC, Chi YY, Mocco JD, Hoh BL (2011) Stent-associated flow remodeling causes further occlusion of incompletely coiled aneurysms. Neurosurgery 69: 598–603 discussion 603–4. 21430583 10.1227/NEU.0b013e3182181c2b

[3] Piotin M, Blanc R, Spelle L, Mounayer C, Piantino R, et al (2010) Stent-assisted coiling of intracranial aneurysms: clinical and angiographic results in 216 consecutive aneurysms. Stroke 41: 110–115. 19959540 10.1161/STROKEAHA.109.558114

[4] Chalouhi N, Jabbour P, Singhal S, Drueding R, Starke RM, et al (2013) Stent-assisted coiling of intracranial aneurysms: predictors of complications, recanalization, and outcome in 508 cases. Stroke 44: 1348–1353. 23512976 10.1161/STROKEAHA.111.000641

[5] Jahshan S, Abla AA, Natarajan SK, Drummond PS, Kan P, et al (2013) Results of stent-assisted vs non-stent-assisted endovascular therapies in 489 cerebral aneurysms: single-center experience. Neurosurgery 72: 232–239. 23149972 10.1227/NEU.0b013e31827b93ea

[6] Geyik S, Yavuz K, Yurttutan N, Saatci I, Cekirge HS (2013) Stent-assisted coiling in endovascular treatment of 500 consecutive cerebral aneurysms with long-term follow-up. AJNR Am J Neuroradiol 34: 2157–2162. 23886748 10.3174/ajnr.A3574 PMC7964833

[7] Hong Y, Wang YJ, Deng Z, Wu Q, Zhang JM (2014) Stent-Assisted Coiling versus Coiling in Treatment of Intracranial Aneurysm: A Systematic Review and Meta-Analysis. PLoS One 9: e82311. 24454690 10.1371/journal.pone.0082311 PMC3893071

[8] Kono K, Terada T (2013) Hemodynamics of 8 Different Configurations of Stenting for Bifurcation Aneurysms. AJNR Am J Neuroradiol 34: 1980–1986. 23578668 10.3174/ajnr.A3479 PMC7965437

[9] Tremmel M, Xiang J, Natarajan SK, Hopkins LN, Siddiqui AH, et al (2010) Alteration of intra-aneurysmal hemodynamics for flow diversion using enterprise and vision stents. World Neurosurg 74: 306–315. 21197155 10.1016/j.wneu.2010.05.008 PMC3011938

[10] Kim M, Levy EI, Meng H, Hopkins LN (2007) Quantification of hemodynamic changes induced by virtual placement of multiple stents across a wide-necked basilar trunk aneurysm. Neurosurgery 61: 1305–12 discussion 1312–3. 18162911 10.1227/01.NEU.0000280168.25968.49 PMC2756037

[11] Cantón G, Levy DI, Lasheras JC (2005) Hemodynamic changes due to stent placement in bifurcating intracranial aneurysms. J Neurosurg 103: 146–155. 16121985 10.3171/jns.2005.103.1.0146

[12] Babiker MH, Gonzalez LF, Ryan J, Albuquerque F, Collins D, et al (2012) Influence of stent configuration on cerebral aneurysm fluid dynamics. J Biomech 45: 440–447. 22226405 10.1016/j.jbiomech.2011.12.016

[13] Gao B, Baharoglu MI, Cohen AD, Malek AM (2012) Stent-assisted coiling of intracranial bifurcation aneurysms leads to immediate and delayed intracranial vascular angle remodeling. AJNR Am J Neuroradiol 33: 649–654. 22194381 10.3174/ajnr.A2841 PMC8050444

[14] Huang QH, Wu YF, Xu Y, Hong B, Zhang L, et al (2011) Vascular geometry change because of endovascular stent placement for anterior communicating artery aneurysms. AJNR Am J Neuroradiol 32: 1721–1725. 21816920 10.3174/ajnr.A2597 PMC7965400

[15] Gao B, Baharoglu MI, Cohen AD, Malek AM (2013) Y-stent coiling of basilar bifurcation aneurysms induces a dynamic angular vascular remodeling with alteration of the apical wall shear stress pattern. Neurosurgery 72: 617–29 discussion 628–9. 23277371 10.1227/NEU.0b013e3182846d9f

[16] Gao B, Baharoglu MI, Malek AM (2013) Angular remodeling in single stent-assisted coiling displaces and attenuates the flow impingement zone at the neck of intracranial bifurcation aneurysms. Neurosurgery 72: 739–48 discussion 748. 23328687 10.1227/NEU.0b013e318286fab3

[17] Kono K, Shintani S, Terada T (2014) Retreatment of Recanalized Aneurysms After Y-stent-assisted Coil Embolization With Double Enterprise Stents: Case Report and Systematic Review of the Literature. Turk Neurosurg 24: 593–597. 25050689 10.5137/1019-5149.JTN.9288-13.0

[18] Kono K, Terada T (2014) Feasibility of insertion of a microcatheter through a Y-stent in coil embolization of cerebral aneurysms and its detailed geometry by micro-computed tomography. Acta Neurochir (Wien) 156: 39–43. 24190455 10.1007/s00701-013-1925-4

[19] Kono K, Shintani A, Tanaka Y, Terada T (2013) Delayed in-stent occlusion due to stent-related changes in vascular geometry after cerebral aneurysm treatment. Neurol Med Chir (Tokyo) 53: 182–185. 23524503 10.2176/nmc.53.182

[20] Kono K, Shintani A, Yoshimura R, Okada H, Tanaka Y, et al (2013) Triple antiplatelet therapy with addition of cilostazol to aspirin and clopidogrel for Y-stent-assisted coil embolization of cerebral aneurysms. Acta Neurochir (Wien) 155: 1549–1557. 23715948 10.1007/s00701-013-1771-4

[21] Kono K, Shintani A, Okada H, Tanaka Y, Terada T (2013) Stent-Assisted Coil Embolization for Cavernous Carotid Artery Aneurysms. Neurol Med Chir (Tokyo) 54: 126–132. 24257503 10.2176/nmc.oa2013-0013 PMC4508706

[22] Kono K, Fujimoto T, Shintani A, Terada T (2012) Hemodynamic characteristics at the rupture site of cerebral aneurysms: a case study. Neurosurgery 71: E1202–E1209. 22922678 10.1227/NEU.0b013e31826f7ede

[23] Kono K, Tomura N, Yoshimura R, Terada T (2013) Changes in wall shear stress magnitude after aneurysm rupture. Acta Neurochir (Wien) 155: 1559–1563. 23715949 10.1007/s00701-013-1773-2

[24] Patel NV, Gounis MJ, Wakhloo AK, Noordhoek N, Blijd J, et al (2011) Contrast-enhanced angiographic cone-beam CT of cerebrovascular stents: experimental optimization and clinical application. AJNR Am J Neuroradiol 32: 137–144. 20966059 10.3174/ajnr.A2239 PMC7964932

[25] Kono K, Shintani A, Fujimoto T, Terada T (2012) Stent-assisted coil embolization and computational fluid dynamics simulations of bilateral vertebral artery dissecting aneurysms presenting with subarachnoid hemorrhage: case report. Neurosurgery 71: E1192–E1201. 22948198 10.1227/NEU.0b013e318270603a

[26] Stuhne GR, Steinman DA (2004) Finite-element modeling of the hemodynamics of stented aneurysms. J Biomech Eng 126: 382–387. 15341176 10.1115/1.1762900

[27] Steinman DA, Hoi Y, Fahy P, Morris L, Walsh MT, et al (2013) Variability of computational fluid dynamics solutions for pressure and flow in a giant aneurysm: the ASME 2012 Summer Bioengineering Conference CFD Challenge. J Biomech Eng 135: 021016. 23445061 10.1115/1.4023382

[28] Ford MD, Alperin N, Lee SH, Holdsworth DW, Steinman DA (2005) Characterization of volumetric flow rate waveforms in the normal internal carotid and vertebral arteries. Physiol Meas 26: 477–488. 15886442 10.1088/0967-3334/26/4/013

[29] Malek AM, Alper SL, Izumo S (1999) Hemodynamic shear stress and its role in atherosclerosis. JAMA 282: 2035–2042. 10591386 10.1001/jama.282.21.2035

[30] Li MH, Gao BL, Fang C, Gu BX, Cheng YS, et al (2006) Angiographic follow-up of cerebral aneurysms treated with Guglielmi detachable coils: an analysis of 162 cases with 173 aneurysms. AJNR Am J Neuroradiol 27: 1107–1112. 16687553 PMC7975718

[31] Kawanabe Y, Sadato A, Taki W, Hashimoto N (2001) Endovascular occlusion of intracranial aneurysms with Guglielmi detachable coils: correlation between coil packing density and coil compaction. Acta Neurochir (Wien) 143: 451–455. 11482694 10.1007/s007010170073

[32] Sluzewski M, van Rooij WJ, Slob MJ, Bescós JO, Slump CH, et al (2004) Relation between aneurysm volume, packing, and compaction in 145 cerebral aneurysms treated with coils. Radiology 231: 653–658. 15118115 10.1148/radiol.2313030460

[33] Chalouhi N, Dumont AS, Hasan D, Tjoumakaris S, Gonzalez LF, et al (2012) Is packing density important in stent-assisted coiling? Neurosurgery 71: 381–6 discussion 386–7. 22569059 10.1227/NEU.0b013e31825c36dd

[34] Kono K, Shintani A, Okada H, Terada T (2013) Preoperative simulations of endovascular treatment for a cerebral aneurysm using a patient-specific vascular silicone model. Neurol Med Chir (Tokyo) 53: 347–351. 23708228 10.2176/nmc.53.347

[35] Mut F, Cebral JR (2012) Effects of flow-diverting device oversizing on hemodynamics alteration in cerebral aneurysms. AJNR Am J Neuroradiol 33: 2010–2016. 22555581 10.3174/ajnr.A3080 PMC7964605

[36] Ma D, Dargush GF, Natarajan SK, Levy EI, Siddiqui AH, et al (2012) Computer modeling of deployment and mechanical expansion of neurovascular flow diverter in patient-specific intracranial aneurysms. J Biomech 45: 2256–2263. 22818662 10.1016/j.jbiomech.2012.06.013

[37] Izar B, Rai A, Raghuram K, Rotruck J, Carpenter J (2011) Comparison of devices used for stent-assisted coiling of intracranial aneurysms. PLoS One 6: e24875. 21966374 10.1371/journal.pone.0024875 PMC3178562

[38] Ford MD, Nikolov HN, Milner JS, Lownie SP, Demont EM, et al (2008) PIV-measured versus CFD-predicted flow dynamics in anatomically realistic cerebral aneurysm models. J Biomech Eng 130: 021015. 18412502 10.1115/1.2900724

[39] Karmonik C, Yen C, Diaz O, Klucznik R, Grossman RG, et al (2010) Temporal variations of wall shear stress parameters in intracranial aneurysms–importance of patient-specific inflow waveforms for CFD calculations. Acta Neurochir (Wien) 152: 1391–8 discussion 1398. 20390310 10.1007/s00701-010-0647-0

[40] Jansen IG, Schneiders JJ, Potters WV, van Ooij P, van den Berg R, et al.. (2014) Generalized versus Patient-Specific Inflow Boundary Conditions in Computational Fluid Dynamics Simulations of Cerebral Aneurysmal Hemodynamics. AJNR Am J Neuroradiol. [Epub ahead of print]. DOI: 10.3174/ajnr.A3901 10.3174/ajnr.A3901 PMC7964445 24651816

[41] Heller RS, Malek AM (2011) Delivery technique plays an important role in determining vessel wall apposition of the Enterprise self-expanding intracranial stent. J Neurointerv Surg 3: 340–343. 21990459 10.1136/jnis.2010.004499 PMC3212648

[42] Ma D, Xiang J, Choi H, Dumont TM, Natarajan SK, et al.. (2014) Enhanced Aneurysmal Flow Diversion Using a Dynamic Push-Pull Technique: An Experimental and Modeling Study. AJNR Am J Neuroradiol. [Epub ahead of print]. DOI: 10.3174/ajnr.A3933 10.3174/ajnr.A3933 PMC7966287 24763414

[43] Ma D, Dumont TM, Kosukegawa H, Ohta M, Yang X, et al (2013) High fidelity virtual stenting (HiFiVS) for intracranial aneurysm flow diversion: in vitro and in silico. Ann Biomed Eng 41: 2143–2156. 23604850 10.1007/s10439-013-0808-4 PMC3766425

[44] Fiorella D, Lylyk P, Szikora I, Kelly ME, Albuquerque FC, et al (2009) Curative cerebrovascular reconstruction with the Pipeline embolization device: the emergence of definitive endovascular therapy for intracranial aneurysms. J Neurointerv Surg 1: 56–65. 21994109 10.1136/jnis.2009.000083

[45] Siddiqui AH, Abla AA, Kan P, Dumont TM, Jahshan S, et al (2012) Panacea or problem: flow diverters in the treatment of symptomatic large or giant fusiform vertebrobasilar aneurysms. J Neurosurg 116: 1258–1266. 22404673 10.3171/2012.2.JNS111942

